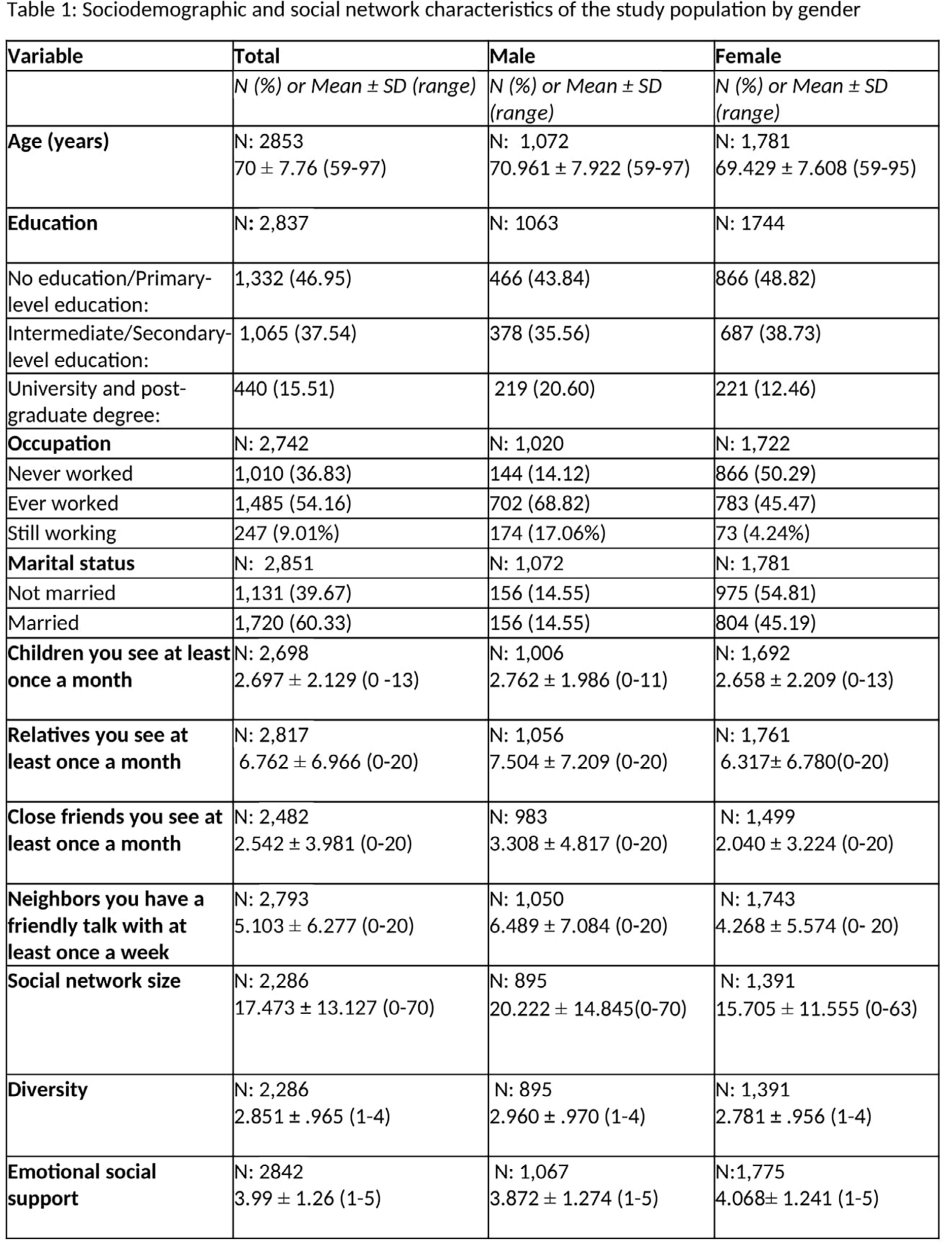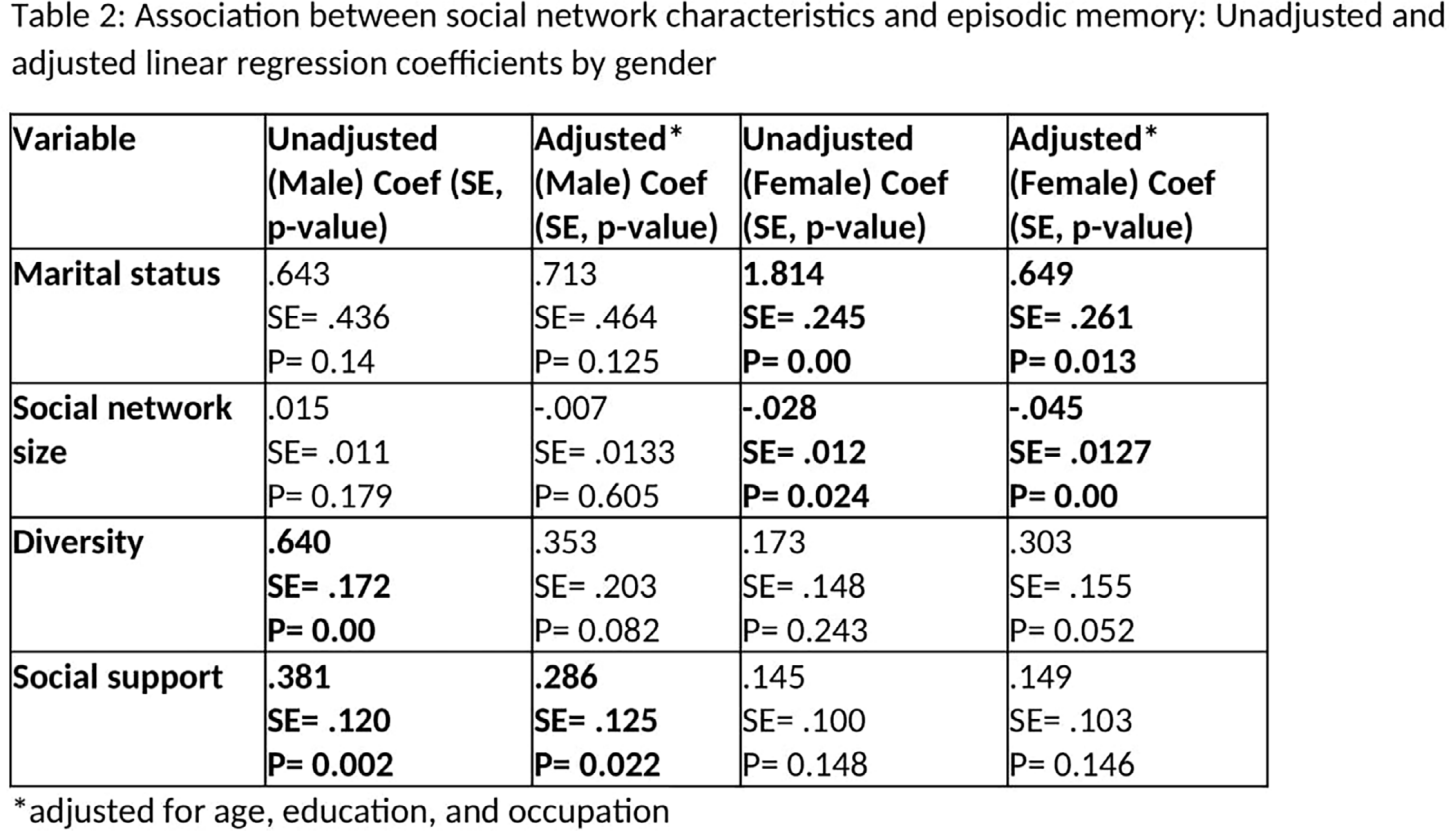# Social networks and cognitive function of older adults in Lebanon: Findings from LSAHA ‐ Lebanon Study on Ageing and Health

**DOI:** 10.1002/alz70860_107214

**Published:** 2025-12-23

**Authors:** Mayssan Kabalan, Sawsan Abdulrahim, Martine Elbejjani, Stephen McCall, Kristine J Ajrouch, Carlos Mendes de Leon, Monique Chaaya

**Affiliations:** ^1^ Faculty of Health Sciences, American University of Beirut, Beirut, Lebanon; ^2^ Faculty of Health Sciences, American University of Beirut, Lebanon, Beirut, Lebanon; ^3^ American University of Beirut, Beirut, Beirut, Lebanon; ^4^ Faculty of Medicine, American University of Beirut, Beirut, Lebanon; ^5^ Center for Research on Population and Health (CRPH), Beirut, Beirut, Lebanon; ^6^ Eastern Michigan University, Ypsilanti, MI, USA; ^7^ Georgetown University, Washington DC, DC, USA; ^8^ Faculty of Health Sciences, American University of Beirut, Beirut, Beirut, Lebanon

## Abstract

**Background:**

Social networks have been linked to cognitive function in later life, yet few studies have explored their association with specific cognitive domains. Among these, episodic memory, crucial for daily functioning, is particularly vulnerable to aging and early cognitive decline. Existing evidence comes primarily from developed countries, with no studies conducted in the Middle East, where social network structures and dynamics may differ. Gender differences in this association also remain unclear, despite evidence that men and women engage with social networks differently. This study aims to examine the association between social network characteristics and episodic memory in older adults in Lebanon, with a particular focus on gender differences.

**Method:**

The study uses baseline data from 2853 participants aged 59+ from LSAHA, a prospective study of older adults in two areas of Lebanon. The social network measure was characterized by its structure (marital status, size, diversity) and function (perceived emotional social support). Network size included children, relatives, close friends, and neighbors with regular contact, while the number of distinct social relationships measured network diversity. Support was assessed using the emotional support subscale of the Arabic‐validated MOS Social Support Survey. Episodic memory was measured using the CERAD 10‐word immediate recall (sum across three trials). Separate linear regressions were conducted for males and females.

**Result:**

Men had larger and more diverse social networks, while women reported greater emotional social support. Greater emotional support was significantly associated with better episodic memory in males (*p* = 0.02). Among females, larger network size was linked to lower episodic memory scores (*p* <0.01), whereas being married was associated with better performance (*p* = 0.013).

**Conclusion:**

The results highlight the role of social networks in cognitive aging and suggest that different network measures (structure versus function) may not benefit cognition for men and women in the same way. This emphasizes the need for tailored approaches in cognitive health interventions. Future research should explore the mechanisms driving these gender differences, including variations in social roles, caregiving, and social support utilization. Additionally, studies should examine the role of social networks in other cognitive domains to enhance specificity and provide a more comprehensive understanding of cognitive aging.